# Dynamic Changes of the Fungal Microbiome in Alcohol Use Disorder

**DOI:** 10.3389/fphys.2021.699253

**Published:** 2021-07-19

**Authors:** Phillipp Hartmann, Sonja Lang, Suling Zeng, Yi Duan, Xinlian Zhang, Yanhan Wang, Marina Bondareva, Andrey Kruglov, Derrick E. Fouts, Peter Stärkel, Bernd Schnabl

**Affiliations:** ^1^Department of Pediatrics, University of California, San Diego, La Jolla, CA, United States; ^2^Department of Medicine, University of California, San Diego, La Jolla, CA, United States; ^3^Department of Medicine, VA San Diego Healthcare System, San Diego, CA, United States; ^4^Division of Biostatistics and Bioinformatics, Department of Family Medicine and Public Health, University of California, San Diego, La Jolla, CA, United States; ^5^Chronic Inflammation Lab, German Rheumatism Research Center, a Leibniz Institute, Berlin, Germany; ^6^Belozerskiy Research Institute for Physical and Chemical Biology and Faculty of Bioengineering and Bioinformatics, M.V. Lomonosov Moscow State University, Moscow, Russia; ^7^Department of Genomic Medicine, J. Craig Venter Institute, Rockville, MD, United States; ^8^Cliniques Universitaires Saint-Luc, Université catholique de Louvain, Brussels, Belgium

**Keywords:** fungi, mycobiome, alcohol-associated liver disease, microbiome, abstinence

## Abstract

**Background:**

Alcohol-associated liver disease (ALD) is an important cause of morbidity and mortality worldwide. The intestinal microbiota is involved in the development and progression of ALD; however, little is known about commensal fungi therein.

**Methods:**

We studied the dynamic changes of the intestinal fungal microbiome, or mycobiome, in 66 patients with alcohol use disorder (AUD) and after 2 weeks of alcohol abstinence using internal transcribed spacer 2 (ITS2) amplicon sequencing of fecal samples.

**Results:**

Patients with AUD had significantly increased abundance of the genera *Candida*, *Debaryomyces*, *Pichia*, *Kluyveromyces*, and *Issatchenkia*, and of the species *Candida albicans* and *Candida zeylanoides* compared with control subjects. Significantly improved liver health markers caspase-cleaved and intact cytokeratin 18 (CK18-M65) levels and controlled attenuation parameter (CAP) in AUD patients after 2 weeks of alcohol abstinence were associated with significantly lower abundance of the genera *Candida*, *Malassezia*, *Pichia*, *Kluyveromyces*, *Issatchenkia*, and the species *C. albicans* and *C. zeylanoides*. This was mirrored by significantly higher specific anti-*C. albicans* immunoglobulin G (IgG) and M (IgM) serum levels in AUD patients in relation to control participants, and significantly decreased anti-*C. albicans* IgG levels in AUD subjects after 2 weeks of abstinence. The intestinal abundance of the genus *Malassezia* was significantly higher in AUD subjects with progressive liver disease compared with non-progressive liver disease.

**Conclusion:**

In conclusion, improved liver health in AUD patients after alcohol abstinence was associated with lower intestinal abundances of *Candida* and *Malassezia*, and lower serum anti-*C. albicans* IgG levels. Intestinal fungi might serve as a therapeutic target to improve the outcome of patients in ALD.

## Background

Alcohol-associated liver disease (ALD) is responsible for 0.9% of all global deaths and 47.9% of all liver cirrhosis-attributable deaths (Rehm et al., 2013), and is the most common indication for liver transplantation in the United States (Cholankeril and Ahmed, 2018). Abstinence is the most important treatment modality in patients with alcohol use disorder (AUD); 4 weeks of abstinence already results in significant improvements of liver stiffness by Fibroscan, aminotransaminases, and gamma-glutamyltransferase (GGT) in patients with AUD (Gianni et al., 2017).

Over the last few years, the contribution of the intestinal bacterial microbiome to liver disease (Hartmann et al., 2012, 2015), and more recently, the role of the fungal microbiome (mycobiome), in liver disease have been investigated (Yang et al., 2017; Bajaj et al., 2018; Chu et al., 2020; Lang et al., 2020; Lemoinne et al., 2020; Jiang et al., 2021). Alcoholic hepatitis (AH) and liver cirrhosis were associated with decreased fungal diversity and increased *Candida* abundance (Bajaj et al., 2018; Lang et al., 2020), whereas primary sclerosing cholangitis showed decreased contributions of *Saccharomyces cerevisiae* (Lemoinne et al., 2020). Although the fungal abundance in the intestine is much lower than the bacterial abundance (Nash et al., 2017), it has been shown that oral treatment with the antifungal amphotericin B reduced intestinal fungal overgrowth, decreased translocation of fungal products into the bloodstream, and ameliorated ethanol-induced liver disease in mice (Yang et al., 2017). It is therefore conceivable that fungi might play a role in ALD. However, studies having investigated the intestinal mycobiome in AUD patients outside the context of AH or advanced cirrhosis are lacking.

The aim of this study was to evaluate the changes of the intestinal mycobiome in AUD and during alcohol abstinence, and how these changes relate to liver disease.

## Materials and Methods

### Patients

Patients with AUD (*n* = 66) were admitted for elective alcohol withdrawal from April 2017 until January 2019 to the alcohol withdrawal unit at St. Luc University Hospital, Brussels, Belgium. They followed a highly standardized and controlled 3-week detoxification and rehabilitation program, which includes a 7-day hospitalization at the start of the 3-week program as well as another 7-day hospitalization during the third week of the program, as described earlier (Maccioni et al., 2020). During hospitalization, the patients received a standard hospital diet, which consisted of 1680 kcal per day with 59 g proteins, 63 g lipids, and 216 g carbohydrates. Patients with antibiotic, probiotic, or prebiotic use during the two months preceding enrollment, immunosuppressive medication, diabetes, inflammatory bowel disease, known liver disease of any other etiology, and clinically significant cardio-vascular, pulmonary or renal co-morbidities were excluded from the study. The use of proton pump inhibitors (PPIs) was not an exclusion criterion but less than 20% of patients used PPIs before inclusion. These subjects were compared to healthy volunteers (*n* = 18) matched for gender, age, and body mass index (BMI) (social drinkers consuming <20 g of alcohol/day). AUD patients were diagnosed by a psychiatrist according to the *Diagnostic and Statistical Manual of Mental Disorders, Fifth Edition (DSM-5)*. All patients received a diagnosis of alcohol dependence (≥6 criteria according to DSM-5). They were heavy drinkers with an alcohol consumption of >60 g/day for more than 1 year (long-term abuse) and actively drinking until the day of admission. More information about psychiatric co-morbidities is provided in the Supplementary Data.

All clinical and biochemical data were collected prospectively from patients, as indicated in the figure and table legends (Maccioni et al., 2020). Collections of biospecimens and imaging were carried out as described elsewhere (Maccioni et al., 2020). In short, on the day of admission, Fibroscan (Echosense, Paris, France) combined with the controlled attenuation parameter (CAP) were performed and a fasting blood sample was drawn. Stool samples were collected from the first bowel movement after admission. Following 2 weeks of abstinence, the Fibroscan was repeated, and paired blood and stool samples were obtained from AUD patients (*n* = 63).

### Ethics Approval and Consent to Participate

As described previously (Maccioni et al., 2020), the study protocol conforms to the ethical guidelines of the 1975 Declaration of Helsinki and was approved by the institution’s human research and ethical committee (Université Catholique de Louvain, Brussels, Belgium; B403201422657). Written informed consent was obtained from all patients and healthy volunteers. We followed the Strengthening the Reporting of Observational Studies in Epidemiology (STROBE) criteria for reporting cohort studies (von Elm et al., 2007).

### Serum Biomarkers

Standard biochemical serum studies, including measurement of aspartate and alanine aminotransferases (AST, ALT), GGT, alkaline phosphatase (AP), were performed at the clinical laboratory associated with St. Luc University Hospital, Brussels, Belgium. Additionally, serum caspase-cleaved and intact cytokeratin 18 (CK18-M65) was used to assess liver cell necrosis and apoptosis (Mueller et al., 2017) (CK18-M65 ELISA kit; TECOmedical AG, Sissach, Switzerland). All assays were performed in duplicate following the manufacturer’s instructions. Antibodies specific to *Candida albicans* in the serum were detected as following: 96-well plates were coated with *C. albicans* lysate (100 μL; 10 μg/mL) overnight at 4°C. After washing with 1× PBST for 30 s, the plates were blocked with 200 μL of 5% PBS/BSA for 1 h at room temperature. Next, plates were washed three times with 200 μL of 1× PBST for 30 s at a time. The serial serum dilutions were prepared in PBS, 100 μL were added to the plate and were incubated overnight at 4°C. After that, plates were washed five times with 200 μL of 1× PBST and detection antibodies were applied: anti-human IgG-AP (ICN/Cappel, Cat No. 59289), anti-human IgM-AP (Sigma, Cat. No. A3437-0.25ML), anti-human IgA-AP (Sigma, Cat. No. A2043) and were incubated for 1 h at 37°C. Subsequently, the plates were washed five times with 200 μL of 1× PBST 100 μL of pNPP (Sigma, Cat. No. N2770) was added to each well. Reactions were stopped by addition of 3M NaOH. Optical densities were measured on Spectramax (Molecular devices). Antibody titers were determined as half-maximal effective concentration (EC50). The EC50 of each sample was calculated by means of a non-linear four parameter regression curve using GraphPad Prism, v.6.0c.

### Fecal DNA Extraction and Fungal Sequencing

Fecal DNA was extracted using the DNA fast stool mini kit (Qiagen, Hilden, Germany) according to the manufacturer’s protocol. Before DNA extraction, bead beating of fecal samples with lysis buffer was performed using 0.7 mm garnet PowerBead tubes (Qiagen, Hilden, Germany). Bead beating was performed using the BioSpec Mini-BeadBeater 96 for 2 × 30 s at 50Hz. PCR and sequencing of the internal transcribed spacer 2 (ITS2) genomic region was performed as previously described using the following primer pair (*italics* = overhang adapter sequence, **bold** = region-specific sequence): 5.8S-Fun (read 1) [*TCGTCGGCAGCGTCAGATGTGTATAAGAGACAG***AACTTT YRRCAAYGGATCWCT**] and ITS4-Fun (read 2) [*GTCTCGT GGGCTCGGAGATGTGTATAAGAGACAG***GCCTCCGCTTATT GATATGCTTAART**] (Taylor et al., 2016), using Illumina’s Fungal Metagenomic Sequencing Demonstrated Protocol^1^. Amplification was performed using KAPA HiFi HotStart ReadyMix (Thermo Fisher Scientific, Waltham, United States). Illumina indices and sequencing adaptors were attached using the Nextera^®^ XT v2 Index Kit following the Illumina ITS SOP. DNA from each sample was pooled into equimolar proportions and sequenced on an Illumina MiSeq platform (PE250) at the University of California, San Diego IGM Genomics Center.

### Bioinformatic Processing of ITS Sequences

CutAdapt v1.8.1 (Martin, 2011) (cutadapt -a ^CCTCCGCT TATTGATATGCTTAART.AGWGATCCRTTGYYRAAAGTT –discard-untrimmed –minimum-length 50 -o trimR2_001.fastq.gz R2_001.fastq.gz) was used to trim amplicon reads of region-specific primer sequences and to discard short reads and reads lacking ITS target primer sequences. Species-level operational taxonomic units (OTUs), clustered at 97% identity, were generated *de novo* from the adapter-trimmed reads using J. Craig Venter Institute’s (JCVI’s) pipeline adaptation of UPARSE (Edgar, 2013; Singh et al., 2019; Freire et al., 2020). Briefly, trimmed R2 sequence reads (from ITS4-fun) were used as input. Sequences of low-quality were discarded and the remaining reads dereplicated prior to determination of abundances. Chimera filtering of the sequences was completed during clustering while taxonomy was assigned to the OTUs with mothur v 1.36.1 (Schloss et al., 2009) using a customized subset of the UNITE fungal ITS database (Nilsson et al., 2019) as the reference (described below). OTUs and corresponding taxonomy assignment tables were generated and used in subsequent analyses. Downstream analyses were performed using the R statistical platform (R Core Team, 2018).

A custom ITS database was generated from the sh_refs_qiime_ver8_97_s_all_04.02.2020 version of the UNITE database that contained both full-length and partial matches to the ITS2 region at least 50 bp in length and only contained taxa known to be host-associated. This was accomplished by first extracting host-associated fungal taxa by selecting genus names matching those in the THF database v1.6.1 (Tang et al., 2015). Full and partial sequences at least 50 bp in length matching the ITS2 region were extracted by running the “host-associated” subset of the UNITE database through ITSx v1.1.2 (Bengtsson-Palme et al., 2013) using the command (ITSx -i sh_refs_qiime_ver8_97_s_all_04.02.2020.THF.fasta -o UNITE_THFdb –cpu 16 –multi_thread T –positions T –not_found T –detailed_results T –partial 49 –save_regions ITS2 –table T). Non-fungal populations detected by ITS2 primers were excluded from final figures.

### Statistics

The Mann–Whitney U test/Wilcoxon rank-sum test was used for non-parametric data (e.g., microbiome data), and results are expressed as median and range for each continuous outcome, if not stated otherwise. The Student’s *t*-test was used for parametric data (e.g., serum markers), and results were expressed as mean and standard deviation for each continuous outcome, if not stated otherwise. The respective statistical test was unpaired for controls versus subjects with AUD, and paired for AUD active versus AUD abstinent. A *P* value equal to or less than 0.05 was considered statistically significant. The fungal sequence reads were normalized to obtain the proportional, relative abundance of each fungus in each patient for further statistical analysis. Principal coordinate analyses (PCoAs) were performed to summarize outcomes of the relative abundance of all fungal genera between the different groups, using unweighted UniFrac (Lozupone et al., 2007). Linear discriminant analysis (LDA) effect size (LEfSe) was used to identify features most likely to account for differences between groups (Segata et al., 2011). Statistical analysis was performed using R statistical software, R version 1.3.1056 for Mac, 2020 the R Foundation for Statistical Computing. To better visualize the changes of relative abundances of fungal subpopulations in particular in the small percentage range in paired analyses of AUD patients before and after abstinence, a “pseudo-log” transformation of the y-axis was performed using the “log_trans” function of the scales library in R (Wickham and Seidel, 2021), since it allows depiction of zero and since it provides a smoother transition around zero (versus linear scale).

## Results

### Study Population With Alcohol Use Disorder

The study population consisted of 66 predominantly male AUD patients and 18 control subjects. Demographic parameters, such as gender, age, and BMI were similar between the groups (Table 1). The majority of AUD patients smoked (80%) whereas only 20% of the controls did. The AST, ALT, GGT, AP, albumin as well as the liver cell necrosis and apoptosis marker caspase-cleaved and intact cytokeratin 18 (CK18-M65) (Mueller et al., 2017) were significantly increased in subjects with AUD compared with controls (Table 1). CK18-M65 with a cutoff value of 400 U/L discriminates progressive (steato-hepatitis, steato-fibrosis) from non-progressive alcohol-ALD (simple steatosis) (Maccioni et al., 2020). Based on Fibroscan evaluation, 84% of the AUD patients had no fibrosis, 10% significant fibrosis and 6% cirrhosis (Supplementary Table 1).

**TABLE 1:** Baseline demographic and laboratory data of the study population.

	Controls (*n* = 18)	Alcohol use disorder (*n* = 66)	*P value*
Gender (male), *n* (%), *n* = 84	14 (78)	47 (71)	0.768
Age (years), *n* = 84	41 ± 12	45 ± 12	0.264
BMI (kg/m^2^), *n* = 84	23.7 ± 3.6	24.4 ± 3.8	0.436
AST (IU/L), *n* = 75	18 ± 5	68 ± 63	<0.001
ALT (IU/L), *n* = 75	11 ± 4	54 ± 41	<0.001
GGT (IU/L), *n* = 74	22 ± 12	207 ± 287	<0.001
AP(IU/L), *n* = 73	48 ± 22	78 ± 33	0.002
Bilirubin (mg/dL), *n* = 75	0.2 ± 0.2	0.6 ± 0.4	<0.001
Albumin (g/dL), *n* = 72	4.4 ± 0.1	4.6 ± 0.4	0.003
INR, *n* = 65	n/a	1 ± 0.1	n/a
Creatinine (mg/dL), *n* = 75	0.97 ± 0.16	0.80 ± 0.14	0.015
Platelets (10^9^/L), *n* = 65	n/a	226 ± 78	n/a
CK18-M65 (U/L), *n* = 113	175.3 ± 65.1	461.3 ± 406.3	<0.001

*Values presented are mean ± standard deviation. The number of subjects for which data were available is indicated in the first column. ALT, alanine aminotransferase; AP, alkaline phosphatase; AST, aspartate aminotransferase; BMI, body mass index; CK18-M65, caspase-cleaved and intact cytokeratin 18; GGT, gamma-glutamyltransferase; INR, international normalized ratio.*

### Changes of the Fungal Composition in Patients With AUD

**FIGURE 1:**
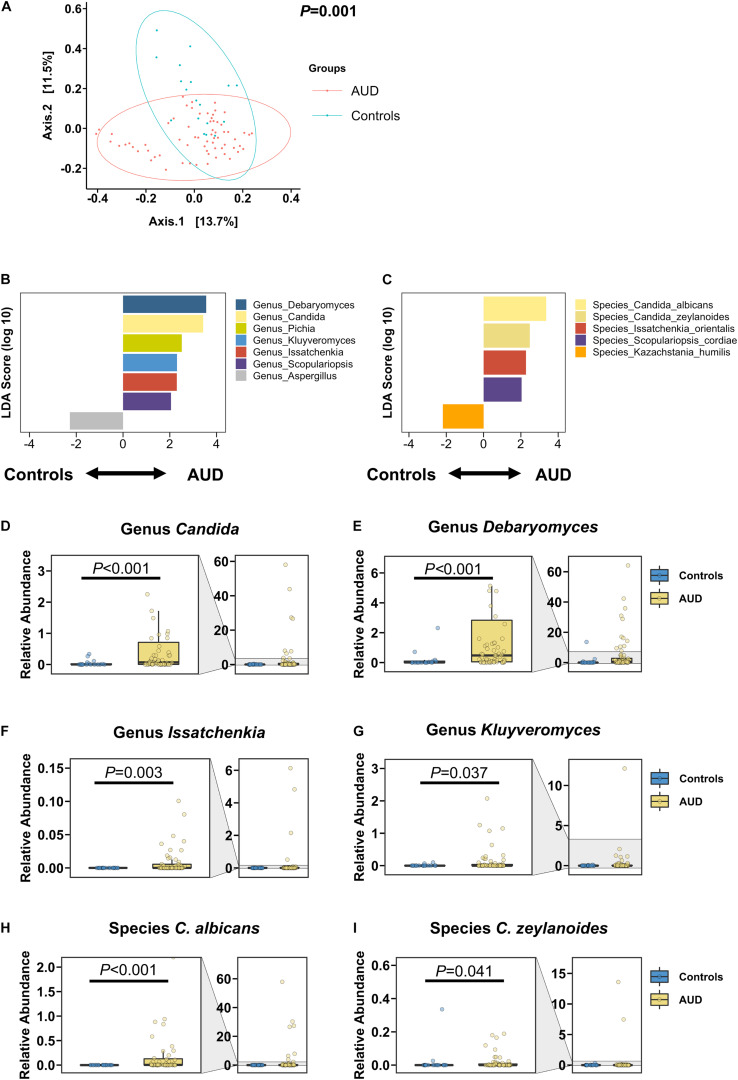
Patients with alcohol use disorder (AUD) have a significantly different mycobiome from control subjects. **(A)** Principal coordinate analysis (PCoA) of mycobiome in AUD patients (*n* = 66) and controls (*n* = 18). **(B,C)** Linear discriminant analysis (LDA) of **(B)** genera and **(C)** species of AUD patients versus controls. **(D–I)** Relative abundance of genera **(D)** *Candida*, **(E)** *Debaryomyces*, **(F)** *Issatchenkia*, **(G)** *Kluyveromyces*, and of species **(H)** *Candida albicans*, and **(I)** *Candida zeylanoides*. A *P* value of equal or less than 0.05 was considered as statistically significant.

The intestinal fungal mycobiome was significantly different in AUD patients in relation to non-alcoholic controls, as shown in the principal coordinate analysis (PCoA; Figure 1A). Based on the LDA effect size, or LEfSe method, the genera *Candida*, *Debaryomyces*, *Pichia*, *Kluyveromyces*, *Issatchenkia*, and *Scopulariopsis* were significantly increased in AUD, whereas the genus *Aspergillus* was significantly decreased in patients with AUD relative to controls. Similarly, AUD patients were found to have significantly higher abundances of the species *C. albicans*, *Candida zeylanoides*, *Issatchenkia orientalis*, and *Scopulariopsis cordiae*, and significantly lower abundances of *Kazachstania humilis* than controls (Figures 1B–I). Further, the families Cystostereaceae, Debaryomycetaceae, Didymellaceae, Microascaceae, and Pichiae were detected at elevated levels in subjects with AUD (Supplementary Figure 1).

### Impact of Abstinence on Fecal Mycobiome in Patients With AUD

**FIGURE 2:**
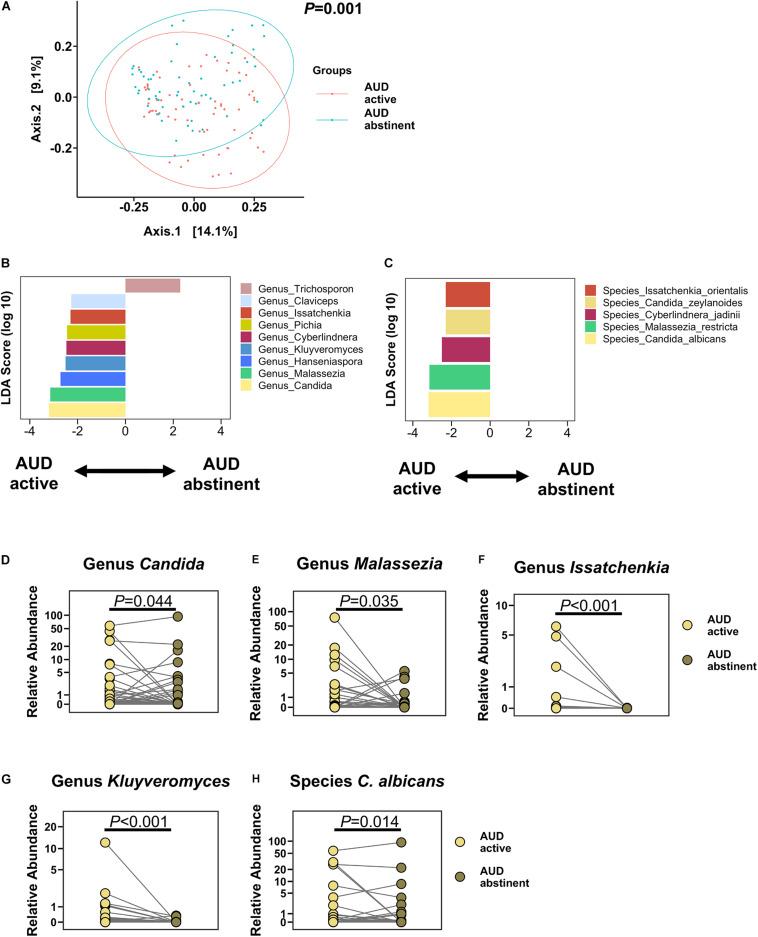
Alcohol abstinence changes the fecal mycobiome. **(A)** PCoA of mycobiome in active AUD patients (*n* = 63) and after abstinence in paired subjects. **(B,C)** LDA of **(B)** genera and **(C)** species of active AUD patients versus after abstinence. **(D–H)** Relative abundance of genera **(D)** *Candida*, **(E)** *Malassezia*, **(F)** *Issatchenkia*, **(G)** *Kluyveromyces*, and of species **(H)** *Candida albicans*. A *P* value of equal or less than 0.05 was considered as statistically significant. AUD, alcohol use disorder; LDA, linear discriminant analysis; PCoA, principal coordinate analysis.

Two weeks of abstinence resulted in significant improvement of liver disease in AUD patients, as measured by significantly lower CK18-M65 levels and decreased hepatic steatosis by controlled attenuation parameter (CAP; Table 2). Abstinence for 2 weeks was not sufficient to change liver stiffness, measured by Fibroscan. Two weeks of abstinence resulted in a significantly different fecal mycobiome profile when compared to active drinkers (Figure 2A). Specifically, *Candida*, *Malassezia*, *Pichia*, *Kluyveromyces*, *Issatchenkia*, *Claviceps*, *Cyberlindnera*, and *Hanseniaspora* were significantly less abundant, whereas *Trichosporon* was significantly enriched in abstinent AUD subjects compared with before alcohol cessation. Abstinence in AUD was associated with significantly lower proportions of the species *C. albicans*, *C. zeylanoides*, *I. orientalis*, and *Cyberlindnera jadinii* than before abstinence (Figures 2B–H). Additionally, the families Saccaromycodaceae, Malasseziaceae, Cystostereaceae, Didymellaceae, and Clavicipitiaceae were detected at significantly more depressed levels in subjects with AUD after abstinence than before abstinence; abstinent AUD subjects had significantly greater levels of the families Metschnikowiaceae and Trichosporonaceae (Supplementary Figure 2).

**TABLE 2:** Imaging and laboratory parameters of AUD patients before and after abstinence.

	AUD active (*n* = 56)	AUD abstinent (*n* = 56)	*P value*
CK18-M65 (U/L, *n* = 52)	461.3 ± 406.3	301.9 ± 251.9	<0.001
CAP (dB/m, *n* = 34)	291.6 ± 60.1	247.8 ± 57.2	<0.001
Stiffness (kPa, *n* = 34)	9.97 ± 12.83	9.56 ± 11.57	0.35

*Values presented are mean ± standard deviation. The number of subjects for which data were available is indicated in the first column. AUD, alcohol use disorder; CAP, controlled attenuation parameter; CK18-M65, caspase-cleaved and intact cytokeratin 18.*

### Differentiation of Progressive Versus Non-progressive Liver Disease Based on Mycobiome

**FIGURE 3:**
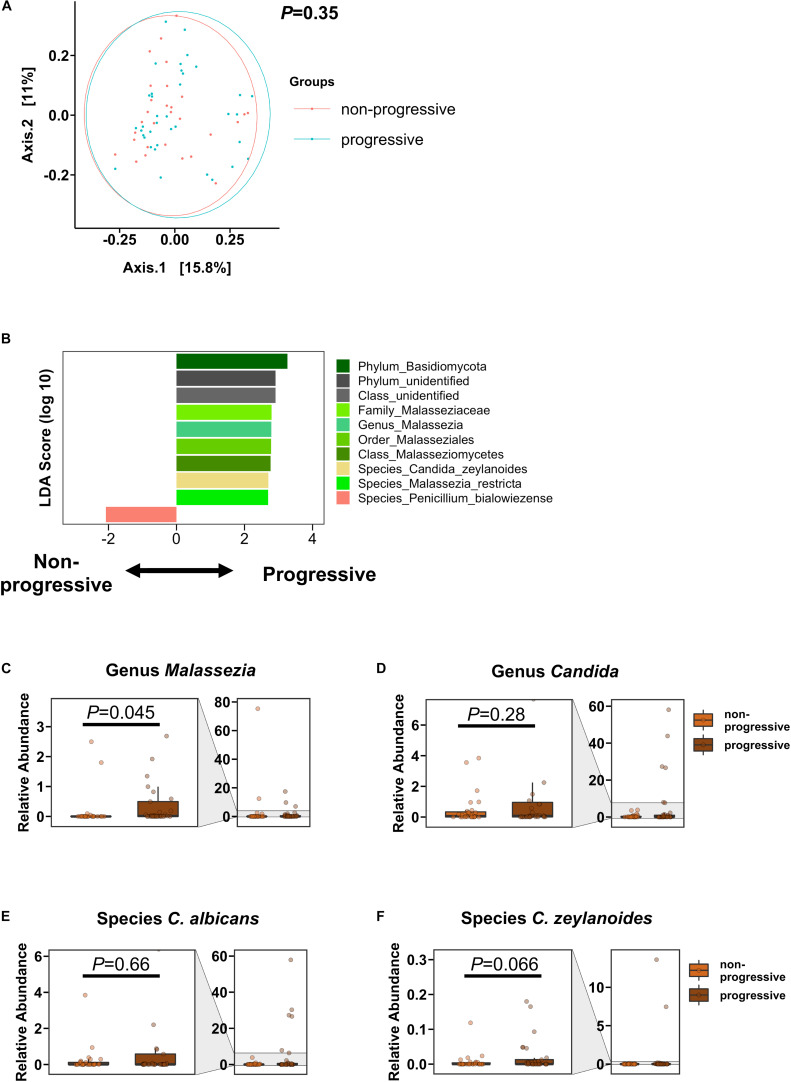
AUD Patients with progressive liver disease have higher abundance of *Malassezia* than AUD patients with non-progressive liver disease. **(A)** PCoA of mycobiome in AUD patients with progressive liver disease (*n* = 37) and AUD patients with non-progressive liver disease (*n* = 29). **(B)** LDA of fungal subpopulations of AUD patients with progressive versus non-progressive liver disease. **(C–F)** Relative abundance of genera **(C)** *Malassezia*, **(D)** *Candida*, and of species **(E)** *Candida albicans*, and **(F)** *Candida zeylanoides*. A *P* value of equal or less than 0.05 was considered as statistically significant. AUD, alcohol use disorder; LDA, linear discriminant analysis; PCoA, principal coordinate analysis.

We defined AUD patients as having progressive liver disease if they had CAP values ≥250 dB/m and AST >40 IU/L, ALT >40 IU/L (steato-hepatitis) and/or fibrosis stage 2 or higher (steato-fibrosis) by Fibroscan (cut-off 7.9 kPa). In the PCoA, the overall mycobiome composition was not significantly different between AUD subjects with progressive versus non-progressive liver disease (Figure 3A). However, the genus *Malassezia* and *Malassezia*-related subpopulations, including the family Malasseziaceae, were significantly increased in subjects with progressive liver disease. *Penicillium bialowiezense* was the only species that was significantly decreased in progressive AUD compared with non-progressive AUD. The genus *Candida* and specifically species *C. albicans* and *C. zeylanoides* were not significantly different in patients with progressive versus non-progressive liver disease, although a trend toward higher abundance of *C. zeylanoides* was noted in the progressive liver disease group (Figures 3B–F).

### Fungal Serum Biomarkers

**FIGURE 4:**
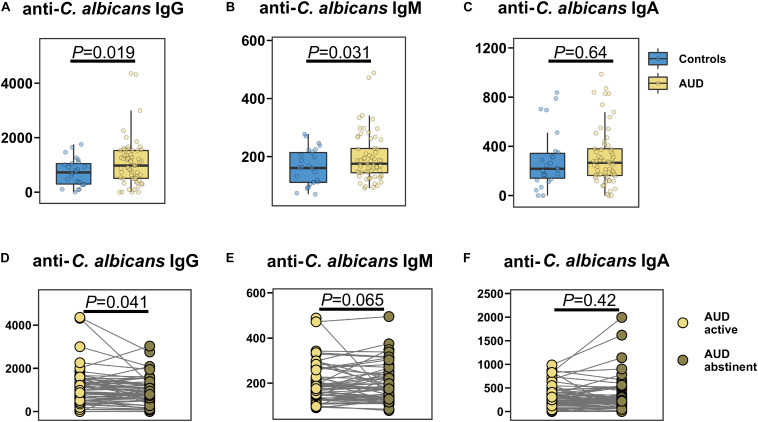
AUD is associated with increased specific anti-*Candida albicans* antibodies in the serum. **(A–C)** Specific anti-*Candida albicans* immunoglobulins (Ig) in AUD patients (*n* = 61) and controls (*n* = 26): **(A)** IgG, **(B)** IgM, **(C)** IgA. **(D–F)** Specific anti-*Candida albicans* Ig in active AUD patients (*n* = 61) and after abstinence in same subjects: **(D)** IgG, **(E)** IgM, **(F)** IgA. A *P* value of equal or less than 0.05 was considered as statistically significant. AUD, alcohol use disorder; Ig, immunoglobulin.

The specific anti-*C. albicans* immunoglobulin G (IgG) and M (IgM) serum levels were significantly higher in AUD patients compared with control participants (Figures 4A,B), whereas anti-*C. albicans* immunoglobulin A (IgA) was similar between the groups (Figure 4C). Abstinence resulted in a significant decrease in anti-*C. albicans* IgG levels, while the anti-*C. albicans* IgM and IgA levels were not significantly different (Figures 4D–F).

## Discussion

To our knowledge, this is the first study to describe dynamic changes of the intestinal mycobiome associated with abstinence in AUD patients. We confirmed that the majority of subjects with AUD have significant liver disease compared with control volunteers, and after 2 weeks of abstinence exhibit a significantly ameliorated liver damage marker CK18-M65 and steatosis marker CAP (both with *P* value < 0.001). Similarly, changes are observed in the intestinal mycobiome: Multiple genera and species are significantly increased in AUD subjects relative to control participants, including *Candida*, *Debaryomyces*, *Pichia*, *Kluyveromyces*, *Issatchenkia*, and *C. albicans* and *C. zeylanoides*. After 2 weeks of abstinence, proportions of the genera *Candida*, *Malassezia*, *Pichia*, *Kluyveromyces*, *Issatchenkia*, and of the species *C. albicans* and *C. zeylanoides* and several more decrease significantly in patients with AUD. Interestingly, the specific anti-*C. albicans* IgG and IgM serum levels are significantly higher in AUD patients compared with control participants, and a short period of abstinence results in a significant decrease in anti-*C. albicans* IgG levels in AUD subjects, mirroring the observed mycobiome changes in the gut. Importantly, the genus *Malassezia* is also discriminatory for progressive and non-progressive liver disease in our AUD cohort.

Patients with AH or liver cirrhosis have markedly elevated proportions of *Candida* (Bajaj et al., 2018; Lang et al., 2020), and *Candida* is the most common cause of fungemia in cirrhotics (Bartoletti et al., 2014) with *C. albicans* being the most common species with a very high 30-day mortality of 35.3% (Bassetti et al., 2017). We show that the intestinal *Candida* abundance as well as the fungal serum biomarker anti-*C. albicans* IgG respond to alcohol abstinence. This suggests that candidemia and its complications could possibly be prevented even in advanced liver disease by strict adherence to alcohol abstinence or by antifungal treatment of *Candida*. However, further studies are required to investigate this hypothesis.

We found a significantly higher contribution of *Malassezia* in progressive liver disease in relation to non-progressive liver disease in our AUD cohort. *Malassezia* is more abundant in patients with inflammatory bowel disease, and might exacerbate disease via inflammatory pathways involving Caspase recruitment domain-containing protein 9 (CARD9; Limon et al., 2019). Similar inflammatory characteristics of *Malassezia* have been described in skin diseases (Sparber et al., 2020). Although a role of *Malassezia* in liver disease has not been established yet, our findings suggest that *Malassezia* with its inflammatory properties might contribute to liver disease in light of the significantly higher contribution in AUD patients with progressive liver disease versus non-progressive liver disease. Interestingly, its contribution also decreased after abstinence, which suggests that alcohol consumption has an impact on the abundance of *Malassezia* and possibly the genus’ impact on disease severity in AUD.

Additionally, the intestinal microbiome, including the mycobiome, is shaped markedly by diet (David et al., 2014). It is hence conceivable that at least part of the findings could be due to the different diet during the hospitalization and the absence of alcohol as opposed to that these changes are due to pathologic changes in the intestinal wall and the liver. Further studies are required in the future to delineate the exact causes for the findings described in this study. Moreover, the samples were obtained in Belgium. As the intestinal mycobiome differs with different geographical locations (Kabwe et al., 2020), and since the intra- and inter-individual variability in fungal abundance is very high (Nash et al., 2017), it is possible that the findings of a follow-up study (in a different country) might differ from the results of the present study.

## Conclusion

In conclusion, patients with AUD have an increased intestinal abundance of *Candida*, which is mirrored by elevated serum anti-*C. albicans* antibodies. Both markers decrease with alcohol abstinence. Anti-*C. albicans* IgG levels might function as a proxy for intestinal *Candida* abundance and might have predictive value in ALD. Intestinal *Malassezia* abundance discriminates between progressive and non-progressive liver disease in AUD. Intestinal fungi might hence serve as a therapeutic target to improve the outcome of patients in ALD. However, confirmation of this assumption requires larger studies in patients with ALD.

## Data Availability Statement

The datasets presented in this study can be found in online repositories. The names of the repository/repositories and accession number(s) can be found below: https://www.ncbi.nlm.nih.gov/, PRJNA703732.

## Ethics Statement

The studies involving human participants were reviewed and approved by the Université Catholique de Louvain, Brussels, Belgium; B403201422657. The patients/participants provided their written informed consent to participate in this study.

## Author Contributions

PS and BS designed the study. PH, SL, XZ, AK, DF, PS, and BS interpreted the data and drafted the work or substantively revised it. All authors developed the method, acquired, analyzed the data, and approved the submitted version and have agreed both to be personally accountable for the author’s own contributions and to ensure that questions related to the accuracy or integrity of any part of the work, even ones in which the author was not personally involved, are appropriately investigated, resolved, and the resolution documented in the literature.

## Conflict of Interest

BS has been consulting for Ferring Research Institute, HOST Therabiomics, Intercept Pharmaceuticals, Mabwell Therapeutics, Patara Pharmaceuticals and Takeda. BS’s institution UC San Diego has received grant support from Axial Biotherapeutics, BiomX, CymaBay Therapeutics, NGM Biopharmaceuticals, Prodigy Biotech and Synlogic Operating Company. The remaining authors declare that the research was conducted in the absence of any commercial or financial relationships that could be construed as a potential conflict of interest.

## Footnotes

1 https://support.illumina.com/downloads/fungal-metagenomic-sequencing-demonstrated-protocol-1000000064940.html

## Abbreviations

AH: alcoholic hepatitis
ALD: alcohol-associated liver disease
ALT: alanine aminotransferase
AP: alkaline phosphatase
AST: aspartate aminotransferase
AUD: alcohol use disorder
BMI: body mass index
CAP: controlled attenuation parameter
CARD9: Caspase recruitment domain-containing protein 9
CK18-M65: caspase-cleaved and intact cytokeratin 18
GGT: gamma-glutamyltransferase
Ig: immunoglobulin
INR: international normalized ratio
ITS2: internal transcribed spacer 2
LDA: Linear discriminant analysis
LEfSe: Linear discriminant analysis effect size
OTUs: operational taxonomic units
PCoA: principal coordinate analysis
STROBE: Strengthening the Reporting of Observational Studies in Epidemiology.
