# Supplementary material for: Dynamic Changes of the Fungal Microbiome in Alcohol Use Disorder
Source: Front Physiol. 2021 Jul 19;12:699253. doi: 10.3389/fphys.2021.699253 (PMC8327211; doi:10.3389/fphys.2021.699253)
Supplement: Supplementary file 1 [file Data_Sheet_1.pdf]

Supplementary data

Psychiatric co-morbidities

A psychiatrist sees all patients at least once in the outpatient clinic before admission. One of the paradigms the alcohol withdrawal is to keep doors open which allows patients to freely circulate within the hospital. Given this open-door policy, patients diagnosed by the psychiatrist with psychiatric comorbidity on axe 1 of the Diagnostic and Statistical Manual-5 or another active addiction, except smoking are not admitted to the unit. Regular monitoring of depression, anxiety and craving is performed in the unit with validated questionnaires (Beck Depression Inventory, State-Trait Anxiety Inventory, Obsessive Compulsive Drinking Scale). The majority of the patients presents with elevated scores at admission that improve with abstinence. Typical mean scores at admission are 22/63 (moderate), 46/63 (severe), 24/40 for depression, anxiety and craving, respectively.

Supplementary Table 1. Fibrosis staging according to Fibroscan.

	No Fibrosis	Significant Fibrosis	Cirrhosis
Cut-off	< 7.6 kPa	≥ 7.6 kPa	≥ 19.5 kPa
N	96	11	7
Median (kPa)	5.1	10.6	24.6
Range (min-max)	3-7.3	8.6-14.9	19.7-72.8

Supplementary Figure 1. AUD significantly impacts the intestinal mycobiome.

LDA of fungal subpopulations in AUD patients (n=66) and controls (n=18). A *P* value of equal or less than 0.05 was considered as statistically significant. AUD, alcohol use disorder; LDA, linear discriminant analysis.

Supplementary Figure 2. Abstinence significantly impacts the intestinal mycobiome in AUD.

LDA of fungal subpopulations in active AUD patients (n=63) and after abstinence in same subjects. A *P* value of equal or less than 0.05 was considered as statistically significant. AUD, alcohol use disorder; LDA, linear discriminant analysis.
