# Supplementary figures and images for: Dynamic Changes of the Fungal Microbiome in Alcohol Use Disorder
Source: Front Physiol. 2021 Jul 19;12:699253. doi: 10.3389/fphys.2021.699253 (PMC8327211; doi:10.3389/fphys.2021.699253)

Supplementary Figure 1

A

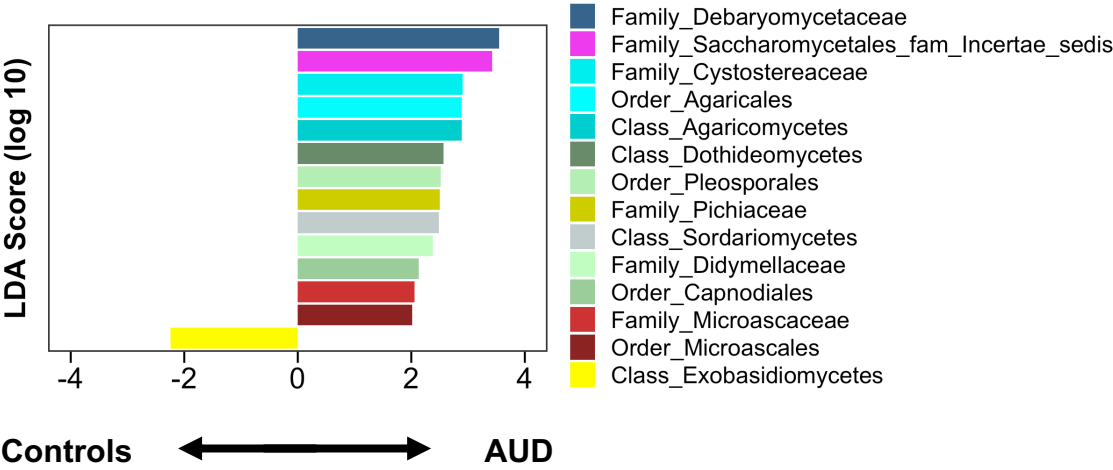

Supplementary Figure 2

A

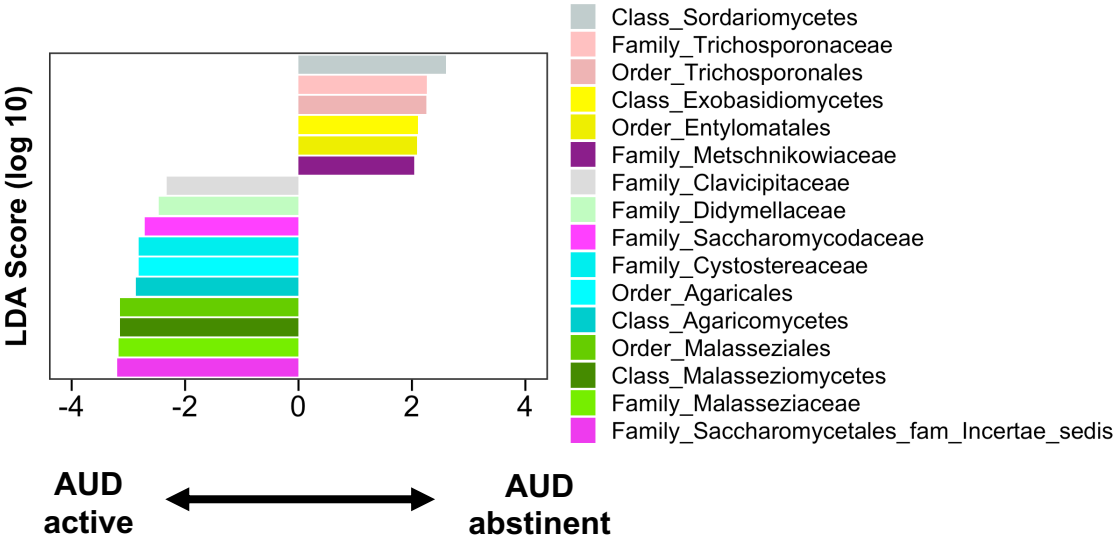

Supplement: Supplementary file 2 [file Data_Sheet_2.pdf]
